# Sound trapping and waveguiding in locally resonant viscoelastic phononic crystals

**DOI:** 10.1038/s41598-023-42452-z

**Published:** 2023-09-15

**Authors:** Kenny L. S. Yip, Sajeev John

**Affiliations:** Department of Physics, University of Toronto, 60 St. George Street, Toronto, ON M5S 1A7 Canada

**Keywords:** Acoustics, Composites

## Abstract

We describe the trapping and absorption of audible sound in centimeter-scale claddings of two-dimensional, locally resonant phononic crystals. In a square lattice of local resonators consisting of steel cores and cellulose shells, embedded in a viscous foam, dual acoustic-range band gaps extending from about 200 to $2850 \text { Hz}$ are achieved. The spectral range consists of a low-frequency, local resonance gap, separated from a higher frequency Bragg resonance gap, by narrow bands of slow-sound modes. We demonstrate that thin claddings of such phononic crystal, of only three unit cells in thickness, can effectively prevent sound transmission, by a combination of reflection and absorption, over much of the audible spectrum. Moreover, frequency-selective sound transmission can be enabled by engineering waveguide channels that transmit sound through the local resonance gap, the Bragg gap, or both. This offers a path to sound-sculpting claddings that can surround a noise-generating source. The viscoelastic foam in our cladding is treated using a fractional Voigt model, capable of describing experimentally observed responses.

## Introduction

The frequency-selective sculpting of audible sound with centimeter-scale cladding material is an endeavor of considerable potential significance^1–3^. The advent of locally resonant acoustic metamaterials has been a vital first step in controlling sound with wavelength scales of meters in air, using centimeter-scale resonators^3–6^. Rapid and accurate fabrication of phononic crystals through recent advances in additive manufacturing has provided the next step forward^7–12^. Phononic crystals, consisting of three-dimensional lattices of centimeter-sized local resonators, can exhibit dual phononic band gaps, extending over a frequency range from 200 to $3700 \text { Hz}$^13,14^. Here, dual phononic band gaps, consisting of separate local resonance and Bragg gaps, can block the transmission of sound through wave-interference effects. The formation of these phononic band gaps may require only a few unit cells in thickness of the periodic structure. When embedded in a realistic viscoelastic foam background, the avenues for sound sculpting involve a combination of local resonance and wave interference. In this article, we present numerical simulation of sound trapping and waveguiding in thin slabs of two-dimensional, locally resonant phononic crystals, toward the goal of audible sound sculpting.

In this paper, we model realistic, open-cell, acoustic foam as a viscoelastic material using the fractional Voigt model^15^. Local resonators typically consist of a dense and stiff core coupled to a stiff shell via a layer of soft interstitial foam. Locally resonant phononic crystals, consisting of an acoustically connected array of such local resonators, typically exhibit two disjoint band gaps, namely a local resonance gap and a Bragg gap. The local resonance gap occurs when the effective mass and moment of inertia of the local resonators are simultaneously negative^13,14,16,17^. On the other hand, the Bragg gap arises from the macroscopic, destructive interference of waves scattered from the lattice.

We illustrate, in two-dimensional examples, that frequency-selective cavity and waveguide modes can be created, respectively, by a point and a line defect in the otherwise periodic lattice. By replacing a local resonator in the lattice by a non-resonant shell, cavity modes which are associated with the translation and rotation of the defect shell are created exclusively in the local resonance gap. On the other hand, the removal of a resonator and subsequent replacement by the background material produce cavity modes exclusively in the Bragg gap.

By replacing a line of local resonators by non-resonant shells, waveguide modes permeate the original local resonance gap, and wave propagation is localized along the line defect via the in-plane translations and rotations of the non-resonant shells. When a line of resonators is removed from the lattice, the local resonance gap remains unaffected. Instead, waveguide modes fill the higher frequency Bragg gap. We show that the frequency spectrum of these waveguides can be controlled by choosing the width of the line defect. By removing a line of the resonators and then reducing the vacant space of foam, a smaller number of waveguide modes can be created and their frequency range can be reduced. By removing a line of resonators and then inserting a solid cellulose rod along the line, we demonstrate that waveguide modes can be engineered simultaneously in both local resonance and Bragg gaps. In a thin cladding of such a phononic crystal, it may be possible to almost completely contain and absorb sound from a noisy source over a broad audible range. By a suitable choice of frequency-selective, waveguide sound outlets that traverse the cladding, it may be possible to sculpt sound almost at will and release a low-intensity, euphonious, version of the sound to the surrounding environment.

## Viscoelasticity in the fractional Voigt model

At a given position $\textbf{x} = (x_{1}, x_{2}, x_{3})$, the field $\textbf{u} = (u_{1}, u_{2}, u_{3})$ defines the displacement from equilibrium of the infinitesimally small parcel of elastic material. The local deformation is given by the symmetric strain tensor $\varepsilon _{ij}$^18^:

1 $$\begin{aligned} \varepsilon _{ij} = \frac{1}{2} \bigg (\frac{\partial u_{i}}{\partial x_{j}} + \frac{\partial u_{j}}{\partial x_{i}} \bigg ). \end{aligned}$$

Linear elasticity is assumed by dropping the nonlinear term $(1/2) \sum _{k} (\partial u_{k} /\partial x_{i}) (\partial u_{k} /\partial x_{j})$.

The energy loss in a damped harmonic oscillator arises from a frictional force that is proportional to the magnitude and opposite in direction to the velocity of the oscillator. Similarly, viscoelasticity in a linear continuum is described by a damping term of the stress that is proportional to the rate of strain^19^:

2 $$\begin{aligned} \sigma _{ij} = C_{ijkl} \varepsilon _{kl} + D_{ijkl} \dfrac{\partial \varepsilon _{kl}}{\partial t}, \end{aligned}$$

where $\sigma _{ij}$ denotes the stress tensor, $C_{ijkl}$ is a fourth order stiffness tensor, and $D_{ijkl}$ is a fourth order damping tensor. The dynamical field equation in a viscoelastic medium of density $\rho$ satisfies Newton’s second law:

3 $$\begin{aligned} \rho \dfrac{\partial ^2 u_{i}}{\partial t^2} = \dfrac{\partial \sigma _{ij}}{\partial x_{j}}. \end{aligned}$$

By forming a scalar product of the dynamical equation and the velocity field, and using the symmetry of the strain tensor, we obtain a continuity equation describing the energy and energy flow densities in the material:

4 $$\begin{aligned} \dfrac{\partial }{\partial t}\left( u_{\text {KE}} + u_{\text {PE}} \right) + \dfrac{\partial S_{i}}{\partial x_{i}} = - p_{\text {loss}}, \end{aligned}$$

where $u_{\text {KE}}$ is the kinetic energy density, $u_{\text {PE}}$ is the elastic potential energy density, $\textbf{S}$ is the energy flux, and $p_{\text {loss}}$ is the power loss density due to viscoelasticity:

5a $$\begin{aligned} u_{\text {KE}}= & {} \dfrac{1}{2} \rho \dfrac{\partial u_{i}}{\partial t} \dfrac{\partial u_{i}}{\partial t}, \end{aligned}$$

5b $$\begin{aligned} u_{\text {PE}}= & {} \dfrac{1}{2} C_{ijkl} \varepsilon _{ij} \varepsilon _{kl}, \end{aligned}$$

5c $$\begin{aligned} S_{i}= & {} - \sigma _{ij} \dfrac{\partial u_{j}}{\partial t}, \end{aligned}$$

5d $$\begin{aligned} p_{\text {loss}}= & {} D_{ijkl} \dfrac{\partial \varepsilon _{ij}}{\partial t} \dfrac{\partial \varepsilon _{kl}}{\partial t}. \end{aligned}$$

In oscillatory systems, it is customary to represent viscoelasticity by complex-valued, effective elastic moduli. At a driving frequency $\omega$, all components oscillate with the time dependence of a complex exponential $e^{-i \omega t}$ in the steady state. By Eq. (2), the damping tensor can be represented by the imaginary part of a generalized, complex-valued stiffness tensor $\tilde{C}_{ijkl}(\omega ) \equiv C_{ijkl} - i \omega D_{ijkl}$. The loss tangent $\eta _{ijkl}(\omega ) \equiv - \text {Im}(\tilde{C}_{ijkl}(\omega ))/\text {Re}(\tilde{C}_{ijkl}(\omega ))$ denotes the ratio of the imaginary part to the real part of the complex-valued stiffness tensor. For simplicity, we assume that the viscoelastic dissipation is isotropic, and is the same for volumetric and shear deformations $\eta _{ijkl} = \eta$.

**Figure 1:**
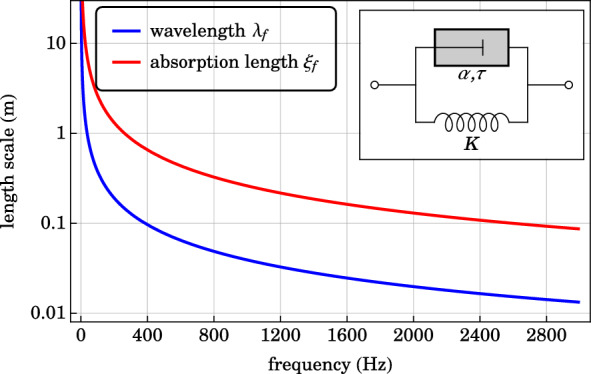
A microscopic fractional Voigt model consists of a spring of spring constant *K*, connected in parallel to a generalized fractional dashpot of power exponent $\alpha$ and characteristic time $\tau$ (see insert). The wavelength $\lambda _{\text {f}}$ (blue) and the absorption length scale $\xi _{\text {f}}$ (red) are plotted in logarithmic scale as a function of frequency. The relevant material constants of the viscoelastic foam are $\rho _{\text {f}} = 25 \text { kg m}^{-3}$, $\tilde{K}(\omega = 0) = 15{,}000 \text { Pa}$, $\alpha = 0.05$ and $\tau = 1 \text { s}$.

The fractional Voigt model for viscoelastic material, depicted in the insert of Fig. 1, consists of an elastic spring connected in parallel to a generalized dashpot^15^. The strain $\varepsilon$ is uniform across the components. The elastic spring of bulk modulus *K* produces a stress $\sigma _{\text {e}} = K \varepsilon$ that is linearly proportional to the applied strain. On the other hand, the generalized dashpot of power exponent $\alpha$ and characteristic time $\tau$ produces a stress $\sigma _{\text {d}} = K (-i \omega \tau )^{\alpha } \varepsilon$. For components connected in parallel, the stresses are additive:

6 $$\begin{aligned} \sigma = \sigma _{\text {e}} + \sigma _{\text {d}} = K \left[ 1 + \left( - i \omega \tau \right) ^{\alpha } \right] \varepsilon , \end{aligned}$$

where a complex-valued, effective modulus $\tilde{K}(\omega )$ and a loss tangent $\eta (\omega )$ are identified:

7a $$\begin{aligned} \tilde{K}(\omega )= & {} K \left[ 1 + \left( - i \omega \tau \right) ^{\alpha } \right] , \end{aligned}$$

7b $$\begin{aligned} \eta (\omega )= & {} \left( \omega \tau \right) ^{\alpha } \sin \left( \pi \alpha /2 \right) /\left[ 1 + \left( \omega \tau \right) ^{\alpha } \cos \left( \pi \alpha /2 \right) \right] . \end{aligned}$$

For positive values of $\alpha$, the effective modulus reduces to the elastic modulus of the elastic spring at zero frequency $\tilde{K}(\omega = 0) = K$. The mechanical response of the generalized dashpot $(\sigma _{\text {d}} = K (-i \omega \tau )^{\alpha } \varepsilon )$ is purely elastic when $\alpha = 0$, and purely viscous when $\alpha = 1$. The generalized fractional dashpot can be interpreted as an analytic interpolation between the purely elastic and purely viscous limits. The strain-stress relation of the generalized dashpot can be formally expressed in terms of Riemann-Liouville fractional calculus^20,21^, and is experimentally shown to accurately characterize soft materials^15,22,23^. For simplicity, we employ the simple fractional Voigt model in this article. More realistic models, such as the fractional Zener model, generally require more springs and generalized dashpots of different power exponents^24,25^.

For a weakly damped foam, we choose $\alpha = 0.05$, $\tau = 1 \text { s}$, $\tilde{K}(\omega = 0) = 15{,}000 \text { Pa}$ and mass density $\rho _{\text {f}} = 25 \text { kg m}^{-3}$. The wavelength $\lambda _{\text {f}} = 2\pi /(\omega \text {Re}[(\rho _{\text {f}}/\tilde{K}(\omega ))^{1/2}])$ and absorption length scale $\xi _{\text {f}} = 1/(\omega \text {Im}[(\rho _{\text {f}}/\tilde{K}(\omega ))^{1/2}])$ are plotted as a function of frequency in Fig. 1. For a phononic crystal structure to exhibit wave-functional properties, the absorption length is at least an order of magnitude longer than the elastic wavelength. This weakly damped regime is referred to as the metamaterial regime in the literature^26^. In this paper, we consider only the weakly damped regime as depicted in Fig. 1. In contrast, when the absorption length is comparable to or shorter than the elastic wavelength, the oscillation is damped in the viscoelastic medium before wave interference has any observable effects. The strongly damped regime is referred to as the boundary layer material regime^26^.

## Locally resonant viscoelastic phononic crystals

Phononic band gaps are observed in locally resonant phononic crystals. The absence of wave modes in a phononic band gap implies that wave propagation is evanescent. The band gaps can be broadly classified into local resonance gaps and Bragg gaps. Local resonance gaps occur over the frequency range where the effective masses and moments of inertia of the acoustically connected local resonators are simultaneously negative. The frequency range of local resonance gaps is insensitive to lattice arrangements, provided that the resonators remain acoustically connected. Bragg gaps are caused by the macroscopic resonances of the array of local resonators, and sensitively depend on the lattice configuration. Frequency-sensitive waveguide modes can be engineered in a desired spectral range, by line defects of local resonance and crystal periodicity.

**Figure 2:**
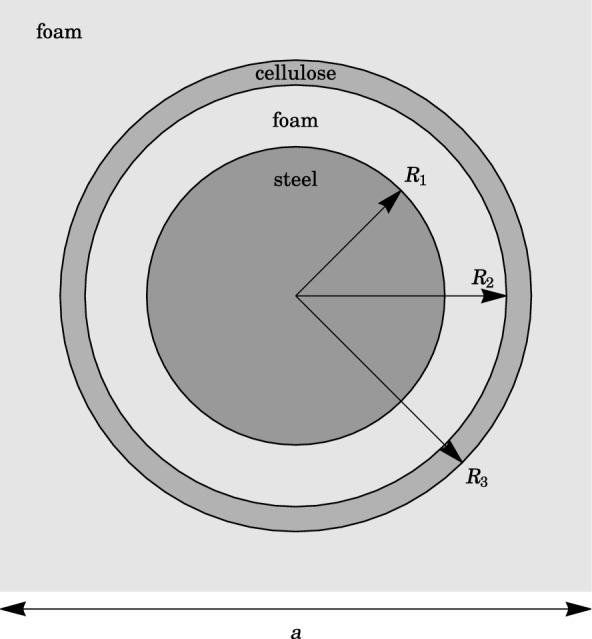
A unit cell of the phononic crystal consists of a dense steel rod of radius $R_{1} \approx 0.2523 \text { cm}$, coupled to a concentric, circular, annular cellulose shell of inner radius $R_{2} \approx 0.3568 \text { cm}$ and outer radius $R_{3} \approx 0.3990 \text { cm}$. The interstitial space and the background are filled with viscoelastic foam. The resonators are arranged in a square lattice of lattice constant $a = 1 \text { cm}$.

The supercell method is employed to study point-defect cavity modes and line-defect waveguide modes in periodic systems. In the present work, we study cavity modes and one-dimensional waveguides in two-dimensional locally resonant phononic crystals. Cylindrically symmetric core-shell resonators are arranged in a square lattice with a lattice constant $a = 1 \text { cm}$. Each local resonator is composed of a dense steel core, coupled to a stiff cellulose shell, via a layer of soft, absorbing foam. The core, interstitial foam and shell constitute $20\%$, $20\%$ and $10\%$ by volume respectively, so that the radius of the core $R_{1} = a \sqrt{0.2/\pi } \approx 0.2523 \text { cm}$, the inner radius of the cellulose shell $R_{2} = a \sqrt{0.4/\pi } \approx 0.3568 \text { cm}$ and the outer radius $R_{3} = a \sqrt{0.5/\pi } \approx 0.3990 \text { cm}$. The background and the interstitial foam are assumed to be the same type of isotropic, fractional Voigt viscoelastic material, with power exponent $\alpha = 0.05$, characteristic time $\tau = 1 \text { s}$, and Lamé constants at zero frequency $\tilde{\lambda }(\omega = 0) = 7500 \text { Pa}$ and $\tilde{\mu }(\omega = 0) = 3750 \text { Pa}$. The mass density and Lamé parameters of steel (cellulose) are $\rho _{\text {c}} = 7940 \text { kg m}^{-3}$, $\lambda _{\text {c}} = 107.5 \text { GPa}$ and $\mu _{\text {c}} = 78.15 \text { GPa}$^27^ ($\rho _{\text {s}} = 1350 \text { kg m}^{-3}$, $\lambda _{\text {s}} = 1.21 \text { GPa}$ and $\mu _{\text {s}} = 0.519 \text { GPa}$^28^). The unit cell is depicted in Fig. 2.

With a small power exponent, the effective elastic moduli are fairly insensitive to frequency at frequencies above $1/\tau$. From $50 \text { Hz}$ to $3000 \text { Hz}$, the real parts of the effective Lamé parameters increase modestly by approximately $13\%$ from $\text {Re}(\tilde{\lambda })(f=50\text { Hz}) \approx 17500 \text { Pa}$ to $\text {Re}(\tilde{\lambda })(f=3000\text { Hz}) \approx 19700 \text { Pa}$, and from $\text {Re}(\tilde{\mu })(f=50\text { Hz}) = 8730 \text { Pa}$ to $\text {Re}(\tilde{\mu })(f=3000\text { Hz}) = 9870 \text { Pa}$. For simplicity, we choose the values at $600 \text { Hz}$ to calculate the supercell band structures and transmission spectra with lossless foam: $\text {Re}(\tilde{\lambda })(f=600\text { Hz}) \approx 18800 \text { Pa}$ and $\text {Re}(\tilde{\mu })(f=600\text { Hz}) = 9390 \text { Pa}$. These particular choices of elastic constants with zero imaginary parts are referred to as lossless foam in this article. The actual band structures and transmission peaks are red-shifted at frequencies below $600 \text { Hz}$ and blue-shifted above $600 \text { Hz}$. As frequency scales inversely with the square root of the elastic moduli, the simplifying assumption introduces about $3.7\%$ error at $50 \text { Hz}$ and $2.4\%$ error at $3000 \text { Hz}$.

The actual band structure with frequency-dependent elastic moduli can be calculated by the Cutting Surface Method^16,29^, where a generalized dispersion $\omega (\textbf{K},\lambda _{\text {p}},\mu _{\text {p}})$ is evaluated parametrically as a function of a hypothetical elastic moduli $\lambda _{\text {p}}$ and $\mu _{\text {p}}$. The resultant dispersion hypersurfaces are cut by the condition $(\lambda _{\text {p}},\mu _{\text {p}}) = (\text {Re}(\tilde{\lambda })(\omega ),\text {Re}(\tilde{\mu })(\omega ))$.

**Figure 3:**
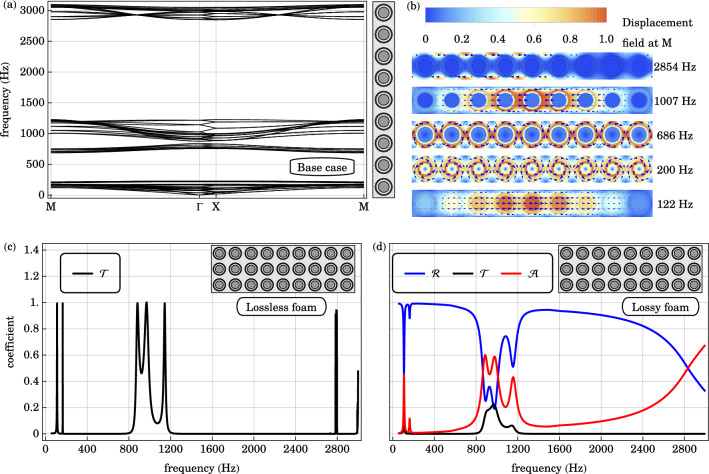
(**a**) The in-plane bands of the $1\times 9$ supercell of a square lattice of cylindrically symmetric core-shell resonators is plotted along a high symmetry path in the irreducible Brillouin zone $\text {M} \rightarrow \Gamma \rightarrow \text {X} \rightarrow \text {M}$. A local resonance gap spans from 217 to $686 \text { Hz}$. The Bragg gap ranges from 1200 to $2850 \text { Hz}$. (**b**) Representative eigenmodes at $\text {M}$ are drawn, which are associated with in-phase translational oscillations at $122 \text { Hz}$, in-phase rotational oscillations at $200 \text { Hz}$, anti-phase rotational oscillations at $686 \text { Hz}$, anti-phase translational oscillations at $1007 \text { Hz}$ and coherent Bragg scattering at $2854 \text { Hz}$. (**c**) The transmission $\mathscr {T}$ spectrum across the lossless, locally resonant, phononic crystal of 3 unit cells in thickness are plotted. (**d**) Similarly, the transmission $\mathscr {T}$, reflection $\mathscr {R}$ and absorption $\mathscr {A}$ coefficients across the locally resonant, viscoelastic, phononic crystal of 3 unit cells in thickness are plotted as a function of frequency. The transmission minima, reflection plateau and absorption minima from 200 to $800 \text { Hz}$, and from to 1300 to $2500 \text { Hz}$ are consistent with the local resonance gap and Bragg gap in the band diagram.

The in-plane bands of the $1\times 9$ supercell are plotted in Fig. 3, complemented with the displacement field profile of representative eigenmodes at the edge of the Brillouin zone $\text {M}$. Each supercell contains nine resonators. Each resonator has a rigid core and a rigid shell, individually providing two in-plane translational degrees of freedom and one in-plane rotational degree of freedom. Therefore, the low-frequency response of the $1\times 9$ supercell is predominantly recaptured by the $9\times 2\times (2+1)=54$ degrees of freedom. The first 27 bands from 0 to $217 \text { Hz}$ are associated with the in-phase translational and rotational oscillations between the cores and shells of individual resonators. These are illustrated by the in-phase translational oscillations at $122 \text { Hz}$ and in-phase rotational oscillations at $200 \text { Hz}$ at $\text {M}$. The 28th to 54th bands from 686 to $1200 \text { Hz}$ are associated with their anti-phase oscillations. At $\text {M}$, the eigenmodes associated with anti-phase rotational and translational oscillations occur at $686 \text { Hz}$ and $1007 \text { Hz}$ respectively. The in-phase and anti-phase bands are separated by a local resonance gap from 217 to $686 \text { Hz}$. Above the anti-phase bands is a Bragg gap from 1200 to $2850 \text { Hz}$. The 55th band occurs above the Bragg gap when the wavelength in the background medium becomes comparable to the lattice spacing. A representative eigenmode at $2854 \text { Hz}$ is drawn. The band structure of the supercell is verified to be consistent with that of a square lattice, with each unit cell containing a single resonator, by band folding.

The finite-element method (FEM) is employed to simulate the reflection, transmission and absorption across a finite-thickness locally resonant phononic crystal. The two-dimensional system is assumed to be infinite in the out-of-plane direction, and repeating in the lateral direction. We consider a cladding that is 3 unit cells thick. The phononic crystal cladding unit cell is chosen to be a $3\times 9$ array of cylindrically symmetric core-shell resonators embedded in foam. We use this large unit cell as our reference for the introduction of defects in subsequent analysis. The phononic crystal slab is sandwiched between two layers of air. At the air-solid interfaces, the boundary conditions are specified by the continuities of normal stress, tangential stress and normal displacement. At solid-solid interfaces within the phononic crystal, both the normal and tangential components of stress and displacement fields are continuous. The two ends of the simulation domain are terminated separately by a perfectly matched layer (PML) with a thickness of several wavelengths. For normal incidence, periodic boundary conditions are imposed on the wavefield on the lateral sides. The fine mesh has at least 21 grid points per wavelength to ensure numerical accuracy.

The transmission, reflection and absorption coefficients across the 3-cm layer of locally resonant acoustic phononic crystal are plotted in Fig. 3. The results are verified to be consistent with the simpler alternative use of a $3\times 1$ array of resonators as the unit cell. The coupling between air and the finite-thickness phononic crystal depends sensitively on the frequency of the incident field, and corresponds to the supercell band diagram in Fig. 3. The reflection troughs, transmission peaks and absorption peaks below $200 \text { Hz}$ are associated with the acoustic bands arising from the in-phase oscillations between the core and the shell. The reflection plateau, flat transmission minimum and absorption minimum from 200 to $800 \text { Hz}$ are related to the local resonance gap. Between 800 to $1200 \text { Hz}$, there are appreciable reflection troughs, and transmission and absorption peaks, which are associated with the anti-phase bands. Over a broad frequency range of the Bragg gap from 1200 to $2400 \text { Hz}$, waves are predominately reflected by the three-unit-cell-thick phononic crystal cladding.

## Sound trapping by frequency-selective cavity modes

The elastic displacement field is evanescent in the band gap of the phononic crystal. Point defects in the otherwise periodic lattice create spatially localized cavity modes, and enable frequency-selective coupling of sound into the band gaps of the original phononic crystal^30,31^. When a resonator is replaced by the background material, cavity modes are created exclusively in the Bragg gap of the original structure. In contrast, removal and subsequent replacement of a resonator by a defect shell creates cavity modes exclusively in the local resonance gap. In the following, we delineate the two types of cavity modes and illustrate their interactions with an incident pressure wave in FEM simulations.

**Figure 4:**
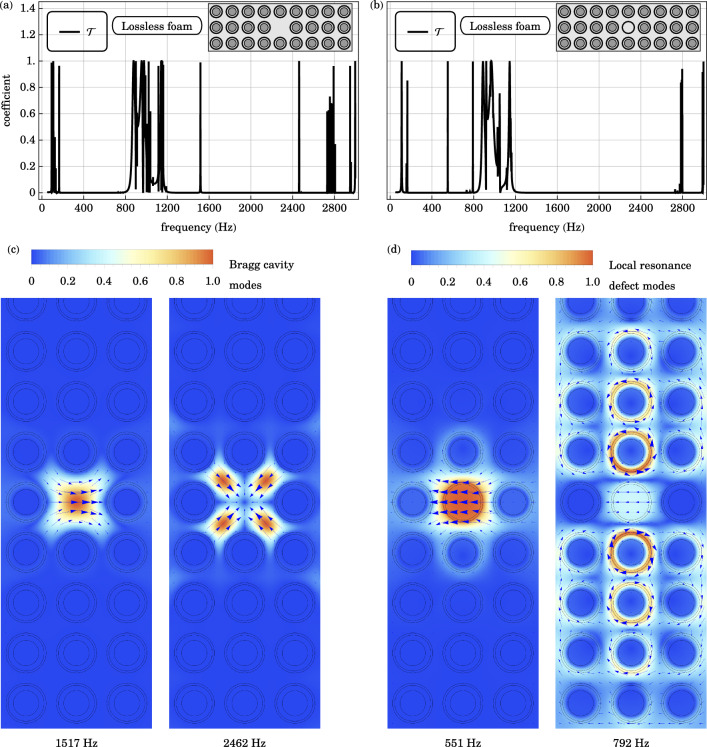
(**a**) The transmission coefficient $\mathscr {T}$ across the locally resonant phononic crystal of 3 unit cells in thickness, with the Bragg point defect, is plotted as a function of frequency. Compared to the base case in Fig. 3, there are additional transmission peaks at $1517 \text { Hz}$ and $2462 \text { Hz}$, associated with dipolar and monopolar cavity modes localized at the positional defect, respectively. (**b**) The transmission coefficient across the locally resonant phononic crystal, with the local resonance point defect, is plotted. The additional transmission peak at $551 \text { Hz}$ in the local resonance gap of the original phononic crystal, is associated with the longitudinal translational oscillation of the defect shell. On the other hand, the transmission peak at $792 \text { Hz}$ is caused by the coupling of the translational oscillation of the defect shell with the anti-phase rotational modes of the adjacent core shell resonators, lying in the anti-phase pass bands of the original phononic crystal. (**c**, **d**) The displacement field profiles of the relevant point defect modes are plotted.

Cavity modes are introduced in the Bragg gap by the removal of a resonator in the otherwise perfect lattice. We refer to this kind of positional disorder as a Bragg point defect. The transmission spectrum across such a locally resonant phononic crystal cladding, of 3 unit cells in thickness, is plotted in Fig. 4. Compared to the base case in Fig. 3, there are additional transmission peaks at $1517 \text { Hz}$ and $2462 \text { Hz}$. These are associated with spatially localized dipolar and monopolar deformations in the background material, respectively, depicted in the lower left panel in Fig. 4. In contrast, the broad transmission minimum over the local resonance band gap from 200 to $700 \text { Hz}$ is unaffected by the Bragg point defect.

Cavity modes are introduced exclusively in the local resonance gap by the replacement of a core-shell resonator by a defect shell. In this case, the steel core of the resonator is replaced by foam. It is referred to as a local resonance point defect. The transmission spectrum across a 3-cm locally resonant phononic crystal slab with a local resonance point defect is plotted in Fig. 4. Compared to the base case in Fig. 3, there are additional transmission peaks at $551 \text { Hz}$ and $792 \text { Hz}$. The elastic displacement fields are depicted in the lower right panel in Fig. 4. The former transmission peak at $551 \text { Hz}$ is associated with the longitudinal translational oscillation of the defect shell. The disturbance is localized near the defect, because wave propagation is evanescent within the local resonance band gap. On the other hand, the latter transmission peak at $792 \text { Hz}$ lies in the frequency range of the anti-phase oscillatory pass bands. The longitudinal translational oscillation of the defect shell is coupled to the anti-phase rotational oscillation of the nearby core-shell resonators. In the original structure, these rotational modes do not couple to the incident pressure field by reflectional and discrete translational symmetries. The defect shell lifts the discrete translational symmetry, enabling excitations of the rotational modes via the translational oscillation of the defect shell. Defect modes can couple effectively to degrees of freedom that are previously inaccessible, unlocking rich and diverse physical responses.

## Frequency-selective waveguides through dual phononic band gaps

Wave propagation is evanescent within the band gaps of a phononic crystal. Frequency-selective transmission of sound can be enabled by engineering specific waveguide channels that allow passage of sound through the otherwise impenetrable phononic band gaps^30,32–37^. Different waveguide designs facilitate sound transmission through parts of the local resonance gap, through parts of the Bragg gap, or both gaps. These involve removal and replacement of local resonators along lines connecting the interior and exterior of the phononic crystal cladding. In the following, we describe the properties of the different types of waveguide modes in viscoelastic, locally resonant, phononic crystal slabs. We also numerically simulate their interactions with an incident pressure wave.

### Bragg waveguides

Waveguide modes are introduced in the Bragg gap by the removal of a line of resonators and re-adjustments of the distance between the phononic crystals on either side of the vacancy. We denote by $d_{\text {wg}}$ the additional distance between the adjacent resonators across the line defect compared to the original lattice. When a line of resonators is removed, $d_{\text {wg}} = a$ and the distance between the centers of the adjacent resonators across the waveguide is $a + d_{\text {wg}} = 2a$.

**Figure 5:**
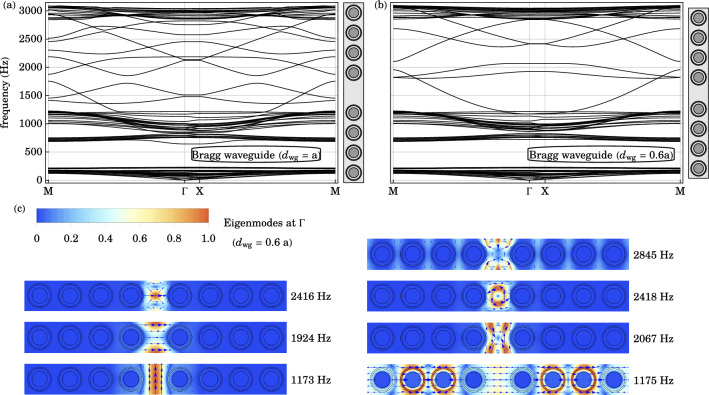
(**a**) The in-plane band structure of the supercell with Bragg waveguide of width $d_{\text {wg}} = a$ is plotted along a high symmetry path in the irreducible Brillouin zone $\text {M} \rightarrow \Gamma \rightarrow \text {X} \rightarrow \text {M}$. (**b**) Similarly, the band structure of the supercell with Bragg waveguide of width $d_{\text {wg}} = 0.6 a$ is plotted. Multiple waveguide bands appear in the Bragg gap of the original structure. The local resonance gap remains almost unaltered by positional disorder. (**c**) For the latter case, the elastic displacement fields of selected eigenmodes, at the Brillouin zone center $\Gamma$, are plotted. The direction of the displacement field is indicated by blue arrows. With a wide waveguide, overtone modes, localized in the line defect, may exist in the Bragg gap.

**Figure 6:**
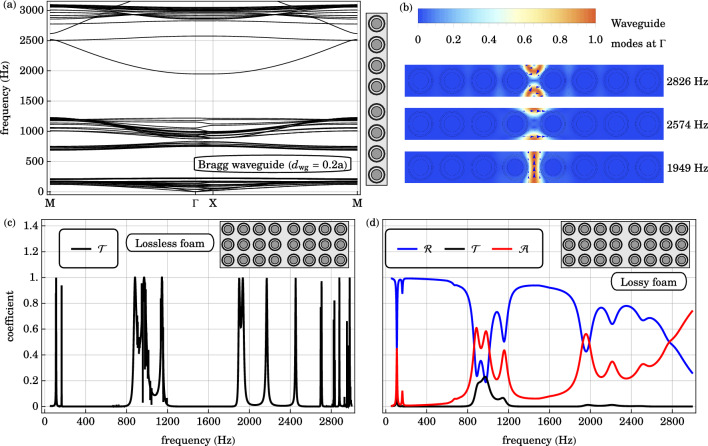
(**a**) The in-plane band structure of the supercell with a Bragg waveguide of width $d_{\text {wg}} = 0.2 a$ is plotted along a high symmetry path in the irreducible Brillouin zone $\text {M} \rightarrow \Gamma \rightarrow \text {X} \rightarrow \text {M}$. Waveguide bands occupy the upper half of the Bragg gap of the original structure. The local resonance gap is insensitive to the slight positional disorder. (**b**) The displacement fields of the waveguide modes, within the Bragg gap, at the Brillouin zone center $\Gamma$, are plotted. The direction of the displacement field is indicated by blue arrows. The waveguide modes are associated with longitudinal oscillation at $1949 \text { Hz}$ (bottom), transverse oscillation at $2574 \text { Hz}$ (middle), and rotational oscillation at $2826 \text { Hz}$ (top). (**c**) The transmission $\mathscr {T}$ spectrum across the lossless, locally resonant, phononic crystal claddings, with a line defect $d_{\text {wg}}=0.2a$, of 3 unit cells in thickness, are plotted. The transmission peaks $1900 \text { Hz}$ to $2600 \text { Hz}$ are associated with the waveguide states within the original Bragg gap. (**d**) Similarly, the transmission $\mathscr {T}$, reflection $\mathscr {R}$ and absorption $\mathscr {A}$ coefficients across the same geometry with the lossless foam replaced by a viscoelastic fractional Voigt material are plotted as a function of frequency. Very small remnants of the transmission peaks in the Bragg gap are still discernible. The reflection plateau and absorption minimum from 200 to $800 \text { Hz}$ arise from the local resonance gap which is insensitive to positional disorder.

The in-plane bands of a supercell with 8 remaining local resonators and a line defect of width $d_{\text {wg}} = a$ is plotted in Fig. 5. Compared to the band structure of the base structure in Fig. 3, numerous bands appear in the original Bragg gap from 1200 to $2850 \text { Hz}$. These waveguide modes occur at practically the same frequencies using larger supercells, confirming the convergence of our supercell method. Similar supercell calculations are repeated with smaller values of $d_{\text {wg}}$. The supercell band diagrams for $d_{\text {wg}} = 0.6 a$ and $d_{\text {wg}} = 0.2a$ are plotted in Figs. 5 and 6 respectively. A striking common feature is the presence of bands in the Bragg gap of the original structure. The number of waveguide modes and their range of frequencies are reduced as $d_{\text {wg}}$ is reduced. On the other hand, the local resonance gap remains unaffected by such line defects.

The waveguide mode elastic displacement fields, for $d_{\text {wg}} = 0.2a$ are plotted in Fig. 6, for sound propagation at the center of the irreducible Brillouin zone $\Gamma$. Arranged in the order of increasing frequency, these additional waveguide modes are associated with the fundamental longitudinal oscillation at $1949 \text { Hz}$, transverse oscillation at $2574 \text { Hz}$ and rotational oscillation at $2826 \text { Hz}$, within the line defect. With a wider line defect, the fundamental modes occur at lower frequencies, while more overtone modes appear within the original Bragg gap. Plotted in Fig. 5 are overtone transverse modes at $1924 \text { Hz}$ and $2416 \text { Hz}$ with one and two nodes, respectively, along the center of the waveguide for $d_{\text {wg}}=0.6a$. In general, with increasing waveguide width, higher harmonics appear and the original Bragg gap is completely filled with propagating sound modes when $d_{\text {wg}} \ge 0.6 a$.

The transmission, reflection and absorption coefficients across the 3-cm cladding with a line defect $d_{\text {wg}} = 0.2 a$ are plotted in Fig. 6. Consistent with the base case in Fig. 3, reflection troughs, transmission peaks and absorption peaks appear over the frequency ranges of the in-phase and anti-phase pass bands. Moreover, the reflection plateau, flat transmision minimum and absorption minimum from 200 to $800 \text { Hz}$ correspond to the local resonance gap, which is insensitive to small amounts of positional disorder. On the other hand, in the case of lossless foam, there are appreciable transmission peaks from 1900 to $2600 \text { Hz}$, within the Bragg gap. Very small remnants of these transmission peaks appear with absorbing foam. With the viscoelastic foam, reflection troughs and absorption peaks coincide with these transmission peaks. These indicate the frequency-selective coupling of the incident acoustic wave with the waveguide modes of the cladding.

The coupling between the incident pressure wave and the phononic crystal cladding is restricted by symmetry requirements and boundary conditions. By reflectional symmetry, the pressure wave at normal incidence can excite the longitudinal waveguide modes, but not the transverse and rotational modes. Moreover, shear and compressional waves propagate at different speeds of sound, and are generally decoupled in linear, isotropic solids. The compressional pressure wave in air does not couple to the transverse and rotational shear waves in the elastic or viscoelastic solids.

### Local resonance defects and waveguides

**Figure 7:**
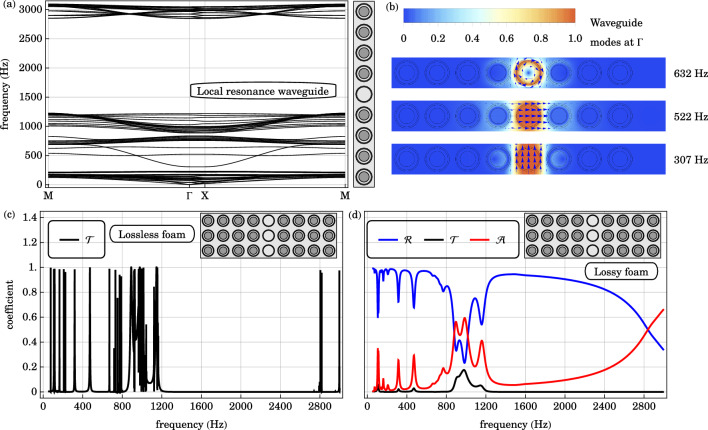
(**a**) The in-plane band structure of the supercell with a local resonance defect waveguide is plotted along a high symmetry path in the irreducible Brillouin zone $\text {M} \rightarrow \Gamma \rightarrow \text {X} \rightarrow \text {M}$. Waveguide bands occupy the local resonance gap of the original structure. The Bragg gap is unaffected by the local resonance defects. (**b**) The elastic displacement fields of the waveguide modes, within the local resonance gap, at the Brillouin zone center $\Gamma$, are plotted. The direction of the displacement field is indicated by blue arrows. The waveguide modes are associated with the longitudinal oscillation of the non-resonant shell at $307 \text { Hz}$ (bottom), its transverse oscillation at $522 \text { Hz}$ (middle), and its rotational oscillation at $632 \text { Hz}$ (top). (**c**) The transmission spectrum $\mathscr {T}$ across a locally resonant, phononic crystal slab with a waveguide line defect of non-resonant shells is plotted. The additional transmission peaks centered at $310 \text { Hz}$ and $460 \text { Hz}$, within the local resonance gap of the original phononic crystal, indicate the coupling of the incident pressure wave with the local resonance waveguide modes. The broad transmission minimum from 1200 to $2800 \text { Hz}$ are consistent with the Bragg gap. (**d**) Similarly, the reflection $\mathscr {R}$, transmission $\mathscr {T}$ and absorption $\mathscr {A}$ coefficients across the viscoelastic phononic crystal slab of the same geometry are plotted as a function of frequency. The reflection dips and absorption peaks from 200 to $800 \text { Hz}$ arise from the waveguide modes along the line defect. These observations agree with the supercell band diagram and waveguide modes.

Waveguide modes are introduced exclusively in the local resonance gap by a line defect where the local resonators are replaced by non-resonant shells. The supercell band structure with 8 local resonators and a line defect of non-resonant shells is plotted in Fig. 7. Compared to the supercell band structure of the base structure in Fig. 3, three additional bands appear in the local resonance gap. On the other hand, the Bragg gap is unaffected by this line defect. This is because the non-resonant shells occupy the same regions as the local resonators in the original structure. At high frequencies relative to the resonant and normal mode frequencies of the local resonator, the effective mass and effective moment of inertia of the core-shell resonator approach the mass and moment of inertia of the shell alone. The relevant waveguide mode displacement fields are plotted in Fig. 7, at the center of the irreducible Brillouin zone $\Gamma$. In the order of increasing frequency, these eigenmodes are associated with the longitudinal oscillation at $307 \text { Hz}$, transverse oscillation at $522 \text { Hz}$ and rotational oscillation at $632 \text { Hz}$, of the defect shell.

The transmission, reflection and absorption coefficients across the 3-cm layer of locally resonant phononic crystal are plotted in Fig. 7. Compared to the base case in Fig. 3, a striking feature is the emergence of reflection troughs, transmission peaks and absorption peaks from 200 to $800 \text { Hz}$, within the frequency range of the local resonance gap. At normal incidence, the pressure wave in air does not couple to the waveguide modes involving the transverse and rotational oscillations of the defects.

### Waveguide with a rigid rod

**Figure 8:**
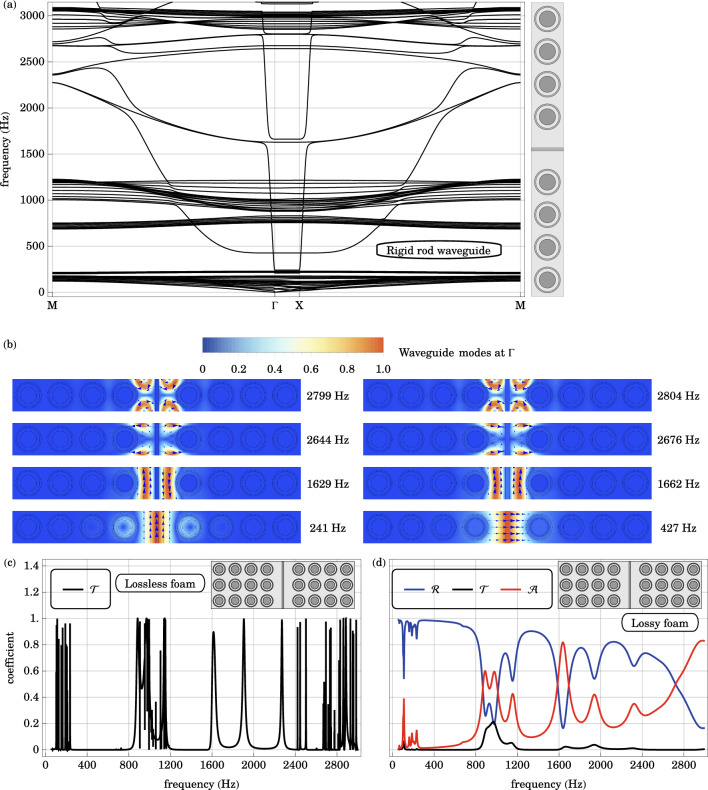
(**a**) The in-plane band structure of the supercell with a rigid rod waveguide is plotted along a high symmetry path in the irreducible Brillouin zone $\text {M} \rightarrow \Gamma \rightarrow \text {X} \rightarrow \text {M}$. Waveguide bands occupy both the local resonance gap and the Bragg gap of the original structure. (**b**) The displacement fields of the waveguide modes at the Brillouin zone center $\Gamma$, are plotted. The direction of the displacement field is indicated by blue arrows. Arranged in the order of increasing frequency from bottom to top, the waveguide modes are associated with the longitudinal oscillation of the solid waveguide at $241 \text { Hz}$, its transverse oscillation at $427 \text { Hz}$, odd-parity longitudinal oscillation of the background foam in the waveguide at $1629 \text { Hz}$, even-parity longitudinal oscillation at $1662 \text { Hz}$, odd-parity transverse oscillation at $2664 \text { Hz}$, even-parity transverse oscillation at $2676 \text { Hz}$, even-parity rotational oscillation at $2799 \text { Hz}$ and odd-parity rotational oscillation at $2804 \text { Hz}$. (**c**) The transmission $\mathscr {T}$ spectrum across a locally resonant, lossless, phononic crystal slab with a rigid rod waveguide is plotted. Compared to the base case, the additional transmission peaks centered at $240 \text { Hz}$, $1600 \text { Hz}$, $1900 \text { Hz}$ and $2300 \text { Hz}$ arise from the longitudinal waveguide modes. (**d**) Similarly, the reflection $\mathscr {R}$, transmission $\mathscr {T}$ and absorption $\mathscr {A}$ coefficients across the same geometry with the viscoelastic foam are plotted as a function of frequency. Reflection dips and absorption peaks occur at frequencies comparable to the transmission peaks in the case with the lossless foam. These observations agree with the supercell band diagram and waveguide modes.

Waveguide modes extend throughout both the local resonance gap and the Bragg gap by the removal of a line of resonators and concomitant addition of a rigid cellulose rod in the waveguide foam. The supercell band structure with 8 local resonators and a line defect with a rigid rod waveguide is plotted in Fig. 8. The cellulose rod, of width 0.1*a*, is made of the same material as the shell of the resonator. Compared to the supercell band structure of the base structure in Fig. 3, additional bands appear in both the local resonance gap and the Bragg gap. The removal of a line of resonators results in propagating sound modes in the Bragg gap of the original phononic crystal. At the same time, the rigid cellulose rod introduces translational oscillation modes within the local resonance gap.

The displacement fields of these relevant waveguide modes are plotted in Fig. 8 at the center $\Gamma$ of the irreducible Brillouin zone. Within the original local resonance gap are two waveguide modes associated with the longitudinal, in-plane translational oscillation of the rigid cellulose rod at $241 \text { Hz}$ and its transverse, in-plane translational oscillation at $427 \text { Hz}$. In-plane rotational oscillation of the rod is inhibited, because the phononic crystal is assumed to be infinite in the longitudinal direction and the relevant planar moment of inertia is theoretically infinite. On the other hand, the Bragg gaps are occupied by odd and even symmetry oscillations in the background foam, on either side of the rigid rod. In the order of increasing frequency, these are the odd-parity longitudinal oscillation at $1629 \text { Hz}$, even-parity longitudinal oscillation at $1662 \text { Hz}$, odd-parity transverse oscillation at $2664 \text { Hz}$, even-parity transverse oscillation at $2676 \text { Hz}$, even-parity rotational oscillation at $2799 \text { Hz}$ and odd-parity rotational oscillation at $2804 \text { Hz}$.

The transmission, reflection and absorption coefficients across the 3-cm layer of locally resonant phononic crystal are plotted in Fig. 8. Compared to the base case in Fig. 3, there are additional reflection dips and absorption peaks in both the local resonance gap and the Bragg gap. Not all waveguide modes, described above, can be excited by an incident pressure wave due to symmetry considerations. The decoupling of shear and compressional waves in isotropic solids inhibits the coupling to the transverse and rotational waveguide modes. At normal incidence, reflection symmetry restricts the excited displacement field in the phononic crystal cladding to be of even parity.

In the above illustrations, overall transmission levels remain quite low due to our use of a relatively lossy background foam. A less lossy, environmentally friendly, natural fiber-reinforced composites^38^ may function quite effectively within locally resonant phononic crystal claddings to selectively allow more transmission of desired sound frequencies.

## Discussion

In summary, we have illustrated the sound trapping and waveguiding properties of various locally resonant phononic crystals. This reveals remarkably broadband control of reflection, transmission and absorption of audible sound in phononic crystal claddings of only a few unit cells in thickness. This is facilitated by the dual band gap nature of the underlying locally resonant medium. Our work reveals important design principles in the construction of acoustic claddings for the mitigation of unwanted noise and the spectral sculpting of sound for the surrounding environment.

Locally resonant phononic crystals exhibit a local resonance gap and a distinct Bragg gap. The former occurs when the effective mass and effective moment of inertia are simultaneously negative and relies on the anti-phase oscillations of acoustically connected resonators. The latter, resulting from the macroscopic resonances of a periodic collection of resonators, scales with the lattice spacing. Spatially localized cavity modes are created in the local resonance gap by replacing a core-shell resonator by a defect shell. Likewise, waveguides in the local resonance gap can be engineered by replacing a line of local resonators by non-resonant shells. The resulting cavity and waveguide modes involve longitudinal, transverse and rotational oscillations of the non-resonant shells. On the other hand, cavity modes appear in the Bragg gap when a core-shell resonator is replaced by the background foam. The frequency extent of the waveguide modes can be controlled by the width of the positional line defect. For a narrow waveguide with $d_{\text {wg}} = 0.2 a$, the waveguide states fill the upper half of the Bragg gap. These correspond to the fundamental longitudinal, transverse and rotational oscillations within the waveguide foam. For a wider waveguide with $d_{\text {wg}} \ge 0.6 a$, the higher harmonics of foam distortion fill the entirety of the Bragg gap.

The acoustic response to an incident pressure wave of the locally resonant phononic crystal that we have considered is predominated by the translational and rotational oscillations of the rigid components. However, the rotational degrees of freedom do not couple effectively when the pressure wave is normally incident. Defects in the phononic crystal lift the discrete translational symmetry of the lattice, enabling richer and more diverse acoustic responses.

Reflection symmetry can be lifted through oblique incidence of the pressure wave. The FEM simulations with the various phononic crystal slabs were repeated at oblique angles of incidence. While the resultant transmission and reflection spectra show small frequency shifts of the transmission peaks, excitations of the rotational modes are insignificant. A plausible explanation is the stark contrast in the speed of sound between air and the phononic crystal constituents. The speed of sound in air is $c_{\text {air}} \approx 343 \text { m s}^{-1}$. The speed of longitudinal disturbance in foam is $c_{\text {f}} = 1/\text {Re}[(\rho _{\text {f}}/\tilde{K})^{1/2}] \approx 40 \text { m s}^{-1}$ at $600 \text { Hz}$. The order of magnitude contrast in the speeds of sound implies that the wave is strongly refracted towards the normal direction, when entering the phononic crystal from air.

In our present work, all numerical simulations are performed in the frequency domain. In principle, considerable computational resources can be saved using time-domain methods. When the finite-thickness structure is excited by a Gaussian wave pulse with a finite spectral width, a broad frequency range of acoustic response can be simulated simultaneously in one trial. The reflection and transmission coefficients over the desired spectral range can be calculated by numerically efficient fast Fourier transform (FFT) of the reflected and transmitted pulses respectively. However, the conventional implementation of finite-difference time-domain (FDTD) method is problematic for structures containing materials with large density contrasts^39^. Future work may focus on numerically stable and accurate implementation of time-domain simulations for elastic and viscoelastic composites involving large contrasts in material parameters. One possibility is to model resonators as rigid bodies with frequency-dependent, effective masses and moments of inertia^13,14,16,17^. Another possibility is employing an adaptive mesh, which is denser in foam with low speeds of sound, and coarse in stiff solids with high speeds of sound. Nevertheless, the mesh in the regions of the stiff solids cannot be too coarse to faithfully represent their geometries.

Future analysis would benefit from more realistic models of environmentally friendly viscoelastic materials. For simplicity, we assumed a simple isotropic fractional Voigt model with identical loss factors for compressional and shear deformations. More realistic models of viscoelastic materials may involve multiple dashpots of different power exponents and characteristic times. The actual loss factors for shear and volumetric deformations may differ, depending on the relative motion between the air in foam and the elastic skeleton^40,41^. Biodegradable “green” materials with less sound absorption are advocated to replace non-recyclable melamine and polyurethane for acoustic panels. These may enable enhanced sound transmission at higher audible frequencies.

An important generalization of our analysis is three-dimensional viscoelastic phononic crystals. It is anticipated that three-dimensional, locally resonant phononic crystals may exhibit an even broader bandwidth of audible sound control. For example, a body-centered cubic crystal of local resonators exhibits dual audible-range band gaps over a frequency range from 600 to $3700 \text { Hz}$^13,14^. The design of waveguide channels in a three-dimensional phononic crystal cladding may provide unprecedented, real-world, opportunities for audible sound control at will.

## Data Availability

The datasets generated during and/or analyzed during the current study are available from the corresponding author upon reasonable request.
